# Chronotherapy With Once-Daily Osilodrostat Improves Cortisol Rhythm, Quality of Life, and Sleep in Cushing's Syndrome

**DOI:** 10.1210/clinem/dgaf206

**Published:** 2025-04-02

**Authors:** Davide Ferrari, Ilaria Bonaventura, Chiara Simeoli, Alessandra Tomaselli, Ludovica Vincenzi, Dario De Alcubierre, Francesca Sciarra, Flavio Rizzo, Lorenzo Cerroni, Nicola Di Paola, Marianna Minnetti, Emilia Sbardella, Mary Anna Venneri, Riccardo Pofi, Rosario Pivonello, Daniele Gianfrilli, Valeria Hasenmajer, Andrea M Isidori

**Affiliations:** Department of Experimental Medicine, “Sapienza” University of Rome, Rome 00161, Italy; Department of Experimental Medicine, “Sapienza” University of Rome, Rome 00161, Italy; Dipartimento di Medicina Clinica e Chirurgia, Sezione di Endocrinologia, Diabetologia, Andrologia e Nutrizione, Università Federico II di Napoli, Naples 80131, Italy; Department of Experimental Medicine, “Sapienza” University of Rome, Rome 00161, Italy; Department of Experimental Medicine, “Sapienza” University of Rome, Rome 00161, Italy; Department of Experimental Medicine, “Sapienza” University of Rome, Rome 00161, Italy; Department of Experimental Medicine, “Sapienza” University of Rome, Rome 00161, Italy; Department of Experimental Medicine, “Sapienza” University of Rome, Rome 00161, Italy; Department of Experimental Medicine, “Sapienza” University of Rome, Rome 00161, Italy; Dipartimento di Medicina Clinica e Chirurgia, Sezione di Endocrinologia, Diabetologia, Andrologia e Nutrizione, Università Federico II di Napoli, Naples 80131, Italy; Department of Experimental Medicine, “Sapienza” University of Rome, Rome 00161, Italy; Department of Experimental Medicine, “Sapienza” University of Rome, Rome 00161, Italy; Department of Experimental Medicine, “Sapienza” University of Rome, Rome 00161, Italy; Oxford Centre for Diabetes, Endocrinology and Metabolism, NIHR Oxford Biomedical Research Centre, University of Oxford, Churchill Hospital, Oxford OX3 7LE, UK; Dipartimento di Medicina Clinica e Chirurgia, Sezione di Endocrinologia, Diabetologia, Andrologia e Nutrizione, Università Federico II di Napoli, Naples 80131, Italy; Department of Experimental Medicine, “Sapienza” University of Rome, Rome 00161, Italy; Department of Experimental Medicine, “Sapienza” University of Rome, Rome 00161, Italy; Department of Experimental Medicine, “Sapienza” University of Rome, Rome 00161, Italy; Centre for Rare Diseases (Endo-ERN Accredited), Policlinico Umberto I, Rome 00161, Italy

**Keywords:** Cushing's syndrome, hypercortisolism, saliva, salivary cortisol, cortisone, cortisol circadian rhythm, chrono-pharmacology, Cushing's Disease, medical therapy

## Abstract

**Context:**

Medical therapy for Cushing syndrome (CS) typically aims to reduce daily cortisol output without addressing circadian rhythm restoration. No licensed drugs target this goal.

**Objective:**

We investigated the efficacy and safety of timed, once-daily osilodrostat administration in improving circadian cortisol profiles in CS.

**Methods:**

A prospective, multicenter study evaluated patients with well-controlled CS on a stable twice-daily osilodrostat therapy before and 60 to 90 days after transitioning to a single equivalent daily dose at 19:00 ± 1 hour. Circadian steroid analysis was performed on saliva, serum, and urine using ultra-high performance liquid chromatography–tandem mass spectrometry. Additional assessments included cardio-metabolic markers, quality of life, sleep function, and safety outcomes.

**Results:**

Sixteen patients (4 males; 7 pituitary, mean age 53.3 ± 11.8 years) were enrolled. At baseline, CS was well-controlled with a mean osilodrostat dose of 4.2 ± 1.3 mg. After transitioning, salivary cortisol exposure decreased significantly during the afternoon to early morning period (AUC_16:00-08:00_: −6.1 [−0.15 to −12.1] ng/mL/h, *P* = .029). Quality of life and sleep improved (CushingQoL: +4.2, *P* = .029; Pittsburgh Sleep Quality Index: −1.7, *P* = .049). Serum steroid precursors, including 11-deoxycorticosterone (−3.1 ng/mL/h, *P* = .008) and 11-deoxycortisol (−17.8 ng/mL/h, *P* = .005), decreased. Eight patients advancing dosing to 16:00 ± 1 hour showed comparable reductions, with phase shifts in acrophase and nadir. No patients developed adrenal insufficiency, liver toxicity, electrocardiogram abnormalities, or loss of disease control.

**Conclusion:**

Once-daily osilodrostat effectively and safely treats patients with biochemically controlled CS, improving circadian cortisol profiles, quality of life, and sleep. Findings support further exploration of chronotherapy-based approaches in CS management.

Cushing syndrome (CS) is a severe endocrine disorder resulting from prolonged exposure to high glucocorticoid levels (1-3). Endogenous hypercortisolism originates from an underlying tumor, most commonly an adrenocorticotropic hormone (ACTH)-secreting pituitary adenoma (Cushing disease [CD], 70%), followed by adrenal cortisol-secreting tumors (20%) and ectopic ACTH or, rarely, corticotropin releasing hormone (CRH)-secreting tumors (10%) (4, 5). CS presents with systemic complications such as impaired glucose and lipid metabolism, obesity, sleep disturbances, cognitive decline, bone loss, sarcopenia, and increased risks of infection and cardiovascular disease (1, 6-12), where disruption of circadian rhythmicity plays a role (13-15). In healthy individuals, cortisol levels rise from 2 to 4 Am, peak shortly after awakening, and decline throughout the day, reaching a nadir around midnight (16). Loss of cortisol's circadian rhythm is a hallmark of CS and contributes to systemic adverse effects by disrupting the coordination of peripheral clock genes (14, 17).

Current guidelines recommend medical therapy to normalize cortisol in patients with persistent or recurrent CS or those unsuitable for surgery (18). However, treatments focus on 24-hour cortisol normalization, assessed by urinary free cortisol (UFC), without addressing circadian rhythm restoration (18). Since endogenous cortisol peaks between 4 Am and 4 Pm (19), elevated evening cortisol levels may go unnoticed with UFC alone. Addressing circadian rhythm is increasingly recognized as a crucial and underexplored research topic, with recent studies highlighting its potential impact on long-term patient outcomes (20, 21). Evidence on cortisol rhythm restoration in CS patients undergoing medical therapy remains limited (22-24), and no validated tools exist to monitor this process. Given the need for a non-invasive, low-stress method to assess circadian profiles, saliva offers a convenient and stable alternative for sample collection, including at home or work. Salivary cortisone is increasingly recognized as a reliable surrogate for serum cortisol, with growing evidence supporting its role in diagnosing and managing adrenal disorders, including adrenal insufficiency and CS (25-29).

Osilodrostat, an 11β-hydroxylase inhibitor, is approved by the European Medicines Agency and the Food and Drug Administration for CS treatment (30, 31). Its remarkable effectiveness has been confirmed in registrative trials, their extensions, several post hoc analyses, and initial real-world experiences, highlighting its ability to lower cortisol levels and relieve CS symptoms and complications (32-41). Osilodrostat primarily acts through reversible, potent CYP11B1 inhibition, reaching peak concentration within 1 hour and having a half-life of ∼4 hours (42). Based on these pharmacokinetics, osilodrostat holds potential for chronotherapy; however, its effects on circadian rhythms and the feasibility of once-daily dosing remain unexplored. Recent in vivo reports from real-world clinical experience suggest that osilodrostat may exert broader effects on the steroidogenic cascade, acting upstream of CYP11B1 and leading to prolonged inhibition persisting for months after treatment discontinuation (43-45), potentially diminishing its impact on circadian rhythm restoration. On these premises, we investigated whether once-daily osilodrostat is a safe and effective alternative for treating CS, aligning steroid secretion with physiological circadian rhythms. Additionally, our study provides a substantial dataset of 24-hour salivary cortisol and cortisone profiles, which could serve as a foundation for developing clinical monitoring tools to evaluate therapeutic adequacy in CS.

## Methods

### Patients

This prospective pilot study included patients enrolled in the multicenter clinical trial investigating circadian rhythms in glucocorticoid disorders (CHROnOS, NCT04374721). Participants were recruited from endocrinology outpatient clinics at “Sapienza” University of Rome and “Federico II” University of Naples between June 2021 and December 2023.

Eligible patients were aged 18 to 80 years with a confirmed diagnosis of endogenous CS, as per the latest guidelines (18). They were either ineligible or unwilling to undergo surgical treatment and were prescribed osilodrostat as a first-line therapy or transitioned from other medical treatments. Inclusion required achieving disease control with UFC levels below the upper limit of normal for at least 1 month on a stable twice-daily osilodrostat regimen. Exclusion criteria included prolonged QTc interval on electrocardiogram (ECG), clinically significant electrolyte imbalances (eg, severe hypokalemia or hypernatremia), or concurrent exogenous glucocorticoid therapy.

### Study Design

Patients were evaluated at baseline (T_BID_) on a stable twice-daily osilodrostat regimen (7:00 and 19:00 ± 1 hour) and again 60 to 90 days after transitioning to an equivalent once-daily evening dose (T_OD_) at 19:00 ± 1 hour. The primary outcome was to compare circadian cortisol rhythms between dosing regimens, focusing on late afternoon to early morning exposure (16:00 to 08:00) and an additional window (16:00 to 04:00) to account for the morning cortisol peak (19, 46). Following an interim safety analysis, a third evaluation (T_OD2_) examined circadian salivary steroid profiles after shifting the once-daily dose to 16:00 ± 1 hour.

Secondary outcomes included patient-reported quality of life and sleep measures, as well as clinical and biochemical assessments of CS comorbidities, such as vital signs, anthropometric measures, and glycolipid metabolism.

### Clinical Examination

Patients underwent comprehensive physical examinations, including measurements of vital signs (arterial blood pressure and heart rate) and anthropometric data (body weight, waist circumference, and body mass index [BMI]). Comorbidities were assessed based on current clinical guidelines: arterial hypertension (47); type 2 diabetes mellitus, impaired fasting glucose, and impaired glucose tolerance (48); dyslipidemia (48); and bone health (osteopenia and osteoporosis) (49).

### Hormonal and Biochemical Assessment

At each visit, blood samples were collected at 08:00 (fasting), 12:00 (pre-lunch), 16:00, and 20:00 (pre-dinner) from a peripheral vein. Saliva samples were obtained via passive drooling (50) at 08:00, 12:00, 16:00, 20:00, and 00:00, according to the most frequently used sampling (17). Additionally, patients collected 24-hour urine samples.

Steroid hormones were analyzed using ultra-high performance liquid chromatography–tandem mass spectrometry (UHPLC-MS/MS) (51). Salivary cortisol and cortisone were measured, while serum samples were analyzed for cortisol, cortisone, 11-deoxycortisol, 11-deoxycorticosterone, and testosterone. Urinary cortisol, cortisone, and testosterone levels were also quantified. Plasma ACTH levels were measured at 08:00 in EDTA plasma samples preserved on ice and analyzed immediately by centrifugation at 2 to 8 °C and immunoradiometric assay, using Beckman Coulter reagents (ref. IM2030, B89463). Since ACTH is very unstable, plasma samples were stored at −20 °C unless the assay was done immediately. The measurement range (from analytical sensitivity to highest calibrator) is 0.31 to approximately 1500 pg/mL. Elevated specificity of the assay was confirmed by extremely low or undetectable cross-reactivity against related molecules (ACTH 1-24; 1-10; 18-39; 11-24; α-melanocyte-stimulating hormone [αMSH], and pro-opiomelanocortin [POMC]) either in the absence (cross-reactivities) or the presence (interferences) of ACTH.

Fasting blood biochemical tests assessed glucose and lipid metabolism (fasting plasma glucose, insulin, glycated hemoglobin [HbA1c], total cholesterol, low-density lipoprotein [LDL], high-density lipoprotein [HDL], triglycerides), liver and kidney function (creatinine, aspartate aminotransferase, alanine aminotransferase, gamma-glutamyl transferase), complete blood count, and electrolytes. Insulin resistance was calculated using homeostatic model assessment (HOMA-IR) ([fasting insulin × fasting glucose] / 405), with a threshold of ≥2.5 indicating resistance (52). Steroid analysis by UHPLC-MS/MS was centralized at Policlinico Umberto I research facility.

### Patient-Reported Measures

Participants completed the CushingQoL questionnaire (53) to evaluate health-related quality of life, the Pittsburgh Sleep Quality Index (PSQI) (54) to assess sleep quality and a Global Assessment Questionnaire to evaluate overall treatment regimen preferences, ease of use in daily routine and future choice.

### Safety Assessments and Adverse Event Monitoring

Safety assessments were conducted monthly from the T_BID_ to T_OD_ regimen. Adrenal insufficiency was monitored through biochemical tests (morning cortisol, fasting glucose, and electrolytes) and weekly telephone check-ins for symptoms of hypoadrenalism. Liver toxicity was assessed via periodic liver panels, while ECGs with QTc measurements were performed at both study centers and evaluated by a cardiologist.

### Statistical Analysis

This exploratory study was designed as a pilot to assess the feasibility, methodology, and potential impact of a once-daily vs a twice-daily dosing regimen of osilodrostat in patients with endogenous CS. The primary objectives were to evaluate circadian hormone profiles, treatment tolerability, and patient-reported outcomes. As a pilot study, the aim was not to test formal hypotheses but to estimate variability in salivary cortisol rhythms (eg, mesor, acrophase, area under the curve [AUC] values) and assess feasibility for future larger-scale studies. Consequently, no formal sample size calculation was performed, consistent with the nature of pilot studies.

The normality of data distribution was assessed using the Shapiro-Wilk test. Continuous variables were reported as mean ± SD or standard error of the mean (SEM) or mean (95% CI) for normally distributed data and as median and interquartile range (IQR, 25%-75%) otherwise. Between-group comparisons were performed using independent samples *t* tests or Mann-Whitney tests, based on data distribution. Differences in binomial proportions for dichotomous variables were analyzed using the chi-square test. Pearson's correlation coefficient was used to estimate correlations between normally distributed continuous variables. Serum steroid daily exposure (08:00 to 20:00) was quantified using the trapezoidal rule to calculate the AUC. Intra-patient changes between timepoints were analyzed using paired samples Student *t* tests or Wilcoxon tests, as appropriate. A *P* value of <.05 was considered statistically significant. Analyses were conducted using SPSS for Windows (v26, SPSS, Inc.), Jamovi, and GraphPad Prism (v10).

### Cosinor-Based Analysis on Salivary Cortisol and Cortisone Curves

Salivary cortisol and cortisone day curves were constructed from samples taken at 08:00, 12:00, 16:00, 20:00, and 00:00. A double harmonic cosinor model was applied to capture the cyclic nature of cortisol secretion, accounting for primary and secondary oscillations, utilizing the Anaconda Python platform for computational analysis. The model is expressed as:


$$\begin{array}{rl} F(x)\;or\;E(x)\;= & M+A1\cdot \cos\;(\frac{2\pi x}{T}-\phi 1)+A2\cdot \cos\;(\frac{4\pi x}{T}-\phi 2) \end{array}$$

where F(x) and E(x) represent the predicted cortisol (F) or cortisone (E) levels at x time; M is the mesor, an estimate of the mean steroid levels, serving as the midpoint around which daily values oscillate; A1 and A2 represent the amplitudes of the first and second harmonic of the model, indicating the primary and secondary fluctuations within the 24-hour cycle; ϕ1 and ϕ2 are the acrophases of the 2 harmonics, meaning the time of the main and secondary daily peaks of the hormone level across the daily cycle; T is the fundamental period of the harmonics, which was set to 24 hours.

This double harmonic model enabled a more detailed characterization of circadian oscillations in steroid levels throughout the day. The first harmonic, representing the primary daily rhythm, is the most significant and captures the main fluctuations in cortisol and cortisone secretion. The second harmonic adds granularity to the model, allowing for a more precise depiction of the complexity of secretion patterns, particularly relevant in this study. Here, fluctuations in cortisol and cortisone levels are influenced by both the underlying disease and the timing differences in osilodrostat administration. To evaluate changes in cortisol and cortisone exposure, the AUC values for salivary cortisol and cortisone during the 16:00-to-08:00 and 16:00-to-04:00 timespans were extracted from the cosinor plots. These timeframes were chosen as they reflect late afternoon to early morning exposure, where improvements would indicate better alignment with the physiological circadian profile. Collectively, for each patient at each timepoint data on mesor, acrophase, amplitude and nadir of the primary harmonic were calculated, as well as the AUC_16:00-08:00_ and AUC_16:00-04:00_ and were compared between T_BID_ and T_OD_ via paired samples T test or Mann-Whitney test based on data distribution.

## Results

### Baseline Characteristics

Between 2021 and 2023, 16 consecutive patients with endogenous CS treated with twice-daily osilodrostat entered the study (mean age 53.3 ± 11.8 years, 12 female). Of these, 7 patients (43.8%) were diagnosed with CD, while 9 (56.3%) had ACTH-independent CS. Among the latter, 4 had a unilateral adrenal adenoma, 1 had bilateral adrenal hyperplasia, 3 presented with bilateral adrenal adenomas, and 1 had bilateral primary macronodular adrenal hyperplasia (BMAH). The median disease duration was 5 years (2-10). Previous surgery was performed in 7 patients: 1 underwent unilateral adrenalectomy for BMAH, and 6 had transsphenoidal surgery for pituitary adenoma. Additionally, 1 patient with CD had prior radiotherapy. Five patients directly transitioned to osilodrostat from a previous medical treatment following a washout of at least 4 weeks (range, 4.4-13.9) to avoid pharmacological interference. Three of these patients shifted from ketoconazole, 1 patient from metyrapone and 1 patient from relacorilant.

At baseline (T_BID_), mean BMI was 29.3 ± 4.4 kg/m^2^, with half of the patients meeting the criteria for overweight and the other half for obesity. Hypertension was prevalent in 14 patients (87.5%), and glucose metabolism impairment was observed in 9 patients (56.3%), including 2 (12.5%) with type 2 diabetes mellitus and 7 (43.8%) with impaired fasting glucose, impaired glucose tolerance, and/or insulin resistance.

The clinical characteristics of the cohort at baseline (T_BID_) are summarized in Table 1. Overall, the majority of patients had mild CS, stably treated with a relatively low dose of osilodrostat (mean 4.2 ± 1.3 mg, range 2-22). Specifically, 9 patients were taking 2 mg/day, 6 patients received between 3 and 7 mg/day, and 1 patient with CD was on 22 mg/day (median dose: 2 mg, IQR_25-75_: 2-4.8). At T_BID_ evaluation, mean osilodrostat treatment duration was 27.7 weeks (range, 8.0-77.6). For the subgroup of 5 patients transitioning from other medical therapies, the mean treatment duration was 59.0 weeks (range, 28.4-77.6).

**Table 1.** Baseline characteristics

	Whole cohort (n = 16)
General characteristics	
Sex (M/F)	4/12
Mean age (years)	53.3 ± 11.8
Disease duration (time from first diagnosis, years)	5 [2-10]
Mean osilodrostat daily dose (mg)	4.2 ± 1.3
CS etiology	
Cushing disease	7 (43.8)
Adrenal CS	9 (56.3)
Previously treated with	
Surgical approach	7 (43.8)
Medical approach	8 (50.0)
Comorbidities	
Overweight	6 (37.5)
Obesity	8 (50.0)
Hypertension	14 (87.5)
Diabetes mellitus (%)	2 (12.5)
Glucose metabolism impairment (IFG/IGT/IR)	7 (43.8)
Dyslipidemia	10 (62.5)
Osteopenia	9 (56.3)
Osteoporosis	2 (12.5)
Fragility fractures	3 (18.8)
Concomitant medications	
1-2 antihypertensive drugs	11 (68.5)
3-4 antihypertensive drugs	3 (18.8)
Antidiabetic drugs	3 (18.8)
Hypolipidemic drugs	7 (43.8)
Antiresorptive drugs	4 (25.0)

Clinical characteristics of the cohort of patients with CS, well-controlled under twice-daily osilodrostat. Continuous variables are expressed as mean ± SD or median [IQR, 25-75] according to distribution while categorical variables as n (%).

Abbreviations: BMI, body mass index; CS, Cushing syndrome; IFG, impaired fasting glucose; IGT, impaired glucose tolerance; IR, insulin resistance.

**Figure 1.**
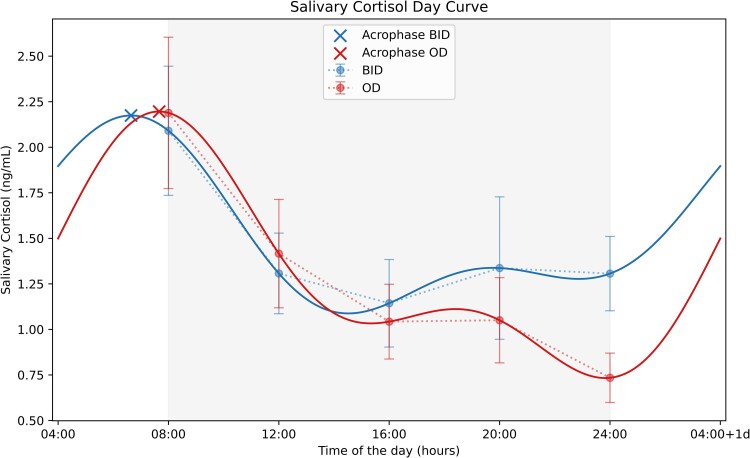
Salivary cortisol curves before and after shifting to once-daily osilodrostat administration. Comparison of T_BID_ and T_OD_ regimens. Data are mean ± SD.

At baseline, salivary cortisol and cortisone day curves revealed similar patterns. Figure 1 illustrates the aggregate salivary cortisol curve for the entire cohort. The acrophase and nadir times were similar between cortisol (acrophase: 06:36 [95% CI: 05:11-08:01]; nadir: 21:03 [95% CI: 18:04-00:03]) and cortisone day curves (acrophase: 06:31 [95% CI: 04:47-08:16]; nadir: 21:45 [95% CI: 15:41-02:16]) (Table 2). The peak-to-trough ratio was considered indicative of daily fluctuation in cortisol and cortisone levels.

**Table 2.** Longitudinal evaluation of hormone profile after osilodrostat shift to evening single administration

	Twice-daily osilodrostat (T_BID_, N = 16)	Once-daily osilodrostat at 19:00 (T_OD_, N = 16)	*P* value
UFC/ULN (nmol/24 hours)	0.59 (0.36-0.74)	0.55 (0.34-0.73)	.820
ACTH (pg/mL)	75.3 (38.0-136.0)	84.3 (43.0-177.0)	.910
Morning cortisol (ng/mL)	112.4 (94.2-130.5)	109.5 (93.5-125.6)	.697
Renin (pg/mL)	19.2 (9.5-35.8)	21.0 (9.0-66.9)	.594
Aldosterone (pg/mL)	100.0 (68.6-131.4)	70.1 (50.2-90.0)	.265
Salivary cortisol profile			
Acrophase time (hh:mm)	06:36 (05:11-08:01)	07:46 (06:31-09:01)	.135
Acrophase level (ng/mL)	1.96 [1.46-3.39]	1.71 [1.03-3.25]	.836
Nadir time (hh:mm)	21:03 (18:04-00:03)	21:03 (16:46-01:19)	.995
Nadir level (ng/mL)	0.42 [0.28-0.82]	0.35 [0.20-0.71]	.918
Mesor (ng/mL)	1.23 [0.93-1.74]	1.03 [0.73-1.39]	.134
Peak-to-trough ratio	4.93 [3.04-9.47]	3.91 [2.64-10.00]	.865
Amplitude (ng/mL)	0.61 [0.28-0.80]	0.47 [0.28-0.75]	1.000
Salivary cortisone profile			
Acrophase time (hh:mm)	06:31 (04:47-08:16)	07:52 (06:25-09:19)	.158
Acrophase level (ng/mL)	15.22 [12.91-22.22]	11.95 [9.40-18.13]	.650
Nadir time (hh:mm)	21:45 [15:41-02:16]	00:00 [22:32-02:22]	.320
Nadir level (ng/mL)	2.99 [0.96-8.38]	2.48 [0.38-6.24]	.683
Mesor (ng/mL)	9.35 [7.57-12.52]	7.55 [5.39-11.57]	.307
Peak-to-trough ratio	3.59 [2.08-6.93]	7.75 [3.24-13.34]	**.033**
Amplitude (ng/mL)	6.73 [2.97-8.61]	4.72 [2.68-7.05]	.650
Serum day curves (8 Am to 8 Pm, ng/mL/h)			
Cortisol AUC	1040.4 [795.5-1331.6]	937.4 [767.4-1153.0]	.191
Cortisone AUC	175.7 (158.5-192.9)	166.6 (146.6-186.6)	.235
DOC AUC	8.47 [7.39-12.46]	5.25 [2.71-10.41]	**.008**
11-deoxycortisol AUC	53.8 [21.41-75.51]	22.6 [15.78-32.91]	**.005**
Testosterone AUC*^a^*	4.33 [2.35-7.40]	3.51 [2.06-4.96]	**.039**
24-hours urine steroids (µg/24 hours)			
Urinary cortisone	10.1 (7.4-12.7)	11.4 (7.0-15.9)	.359
Urinary testosterone*^a^*	0.93 (0.51-1.36)	0.91 (0.38-1.43)	.998

Data are expressed as mean (IC95) or median [IQR, 25-75] according to distribution. Values in bold indicate statistically significant results (*P* < .05)

Abbreviations: ACTH, adrenocorticotropic hormone; AUC, area under the curve; DOC, 11-deoxycorticosterone; UFC, urinary free cortisol; ULN, upper limit of normal.

^*a*^Reported only for female patients.

### Chronopharmacological Intervention

All enrolled patients completed the study protocol, and the main results of the longitudinal evaluation are presented in Table 2 and Table 3, while questionnaire scores are detailed in Table 4.

**Table 3.** Longitudinal evaluation of anthropometric parameters and metabolism following the shift to once-daily, evening osilodrostat

	Twice-daily osilodrostat (T_BID_) N = 16	Once-daily osilodrostat at 19:00 (T_OD_) N = 16	*P* value
Vital signs and anthropometric measures			
BMI (kg/m^2^)	29.3 (26.9-31.6)	29.3 (27.3-31.3)	.174
Waist circumference (cm)	104.0 (96.2-112.0)	103.5 (97.7-109.3)	.209
Visceral adiposity index	5.29 (3.32-7.26)	5.31 (2.89-7.74)	.185
Systolic BP (mmHg)	133 (122-145)	131 (120-141)	.669
Diastolic BP (mmHg)	88 (80-95)	83 (77-88)	.234
Mean BP (mmHg)	104 (96-111)	98 (92-104)	.164
Pulse rate (bpm)	76 (71-81)	75 (70-80)	.684
Glycolipid metabolism			
Fasting plasma glucose (mg/dL)	86.6 (78.3-95.0)	84.0 (73.3-94.8)	.820
Fasting insulin (µUI/mL)	10.4 [5.0-17.2]	11.3 [7.0-14.0)	.333
HbA1c (%)	5.6 (5.4-5.8)	5.5 (5.3-5.8)	.838
HOMA index	1.78 [1.02-3.53]	1.45 [1.16-2.7]	.594
Total cholesterol (mg/dL)	181 (164-197)	166 (140-192)	.344
HDL cholesterol (mg/dL)	43 [39-52]	47 (40-64)	.169
LDL cholesterol (mg/dL)	101 [83-115]	91 [77-110]	.328
Triglycerides (mg/dL)	127 (97-157)	115 (83-147)	.268
Safety lab assessments			
Sodium (mmol/L)	141.9 (140.9-142.8)	141.4 (139.9-142.8)	.236
Potassium (mmol/L)	4.4 (4.2-4.7)	4.4 (4.1-4.7)	.543
GOT (U/L)	17.5 [15.0-25.0]	20.0 [14.75-26.25]	.801
GPT (U/L)	22.0 [11.3-30.3]	20.0 [12.75-30.25]	.484
γ-GT (U/L)	20.0 [14.5-46.8]	16.0 [11.5-20.0]	.446

Data are expressed as mean (IC95) or median [IQR, 25-75] according to distribution.

Abbreviations: BMI, body mass index; BP, blood pressure; GOT, aspartate aminotransferase; GPT, alanine aminotransferase; γ-GT, gamma-glutamyl transferase; HbA1c, glycated hemoglobin; HDL, high-density lipoproteins; HOMA, homeostatic model assessment; LDL, low-density lipoproteins.

**Table 4.** Longitudinal evaluation of quality of life and sleep after osilodrostat shift to once-daily, evening administration

	Twice-daily osilodrostat (T_BID_)	Once-daily osilodrostat (T_OD_)	*P* value
Sleep quality			
PSQI total score	9.9 ± 4.7	8.2 ± 4.8	**.049**
PSQI 1 (subjective sleep quality)	1.5 ± 1.1	1.8 ± 1.1	.804
PSQI 2 (sleep latency)	1.4 ± 1.3	0.9 ± 1.1	**.024**
PSQI 3 (sleep duration)	1.8 ± 1.2	1.1 ± 1.1	**.024**
PSQI 4 (sleep efficiency)	0.9 ± 1.1	0.7 ± 1.0	.295
PSQI 5 (sleep disturbance)	1.9 ± 0.6	1.7 ± 0.8	.212
PSQI 6 (use of sleep medication)	1.5 ± 1.5	0.8 ± 1.3	.085
PSQI 7 (daytime dysfunction)	1.4 ± 0.9	1.2 ± 1.0	.283
Quality of life			
CushingQoL total score	43.7 ± 15.9	47.9 ± 17.6	**.029**

Data are expressed as mean ± SD. Statistically significant values are shown in bold.

Abbreviations: PSQI, Pittsburgh Sleep Quality Index.

### Changes in Salivary Cortisol Profile With Once-daily Osilodrostat Administration

**Figure 2.**
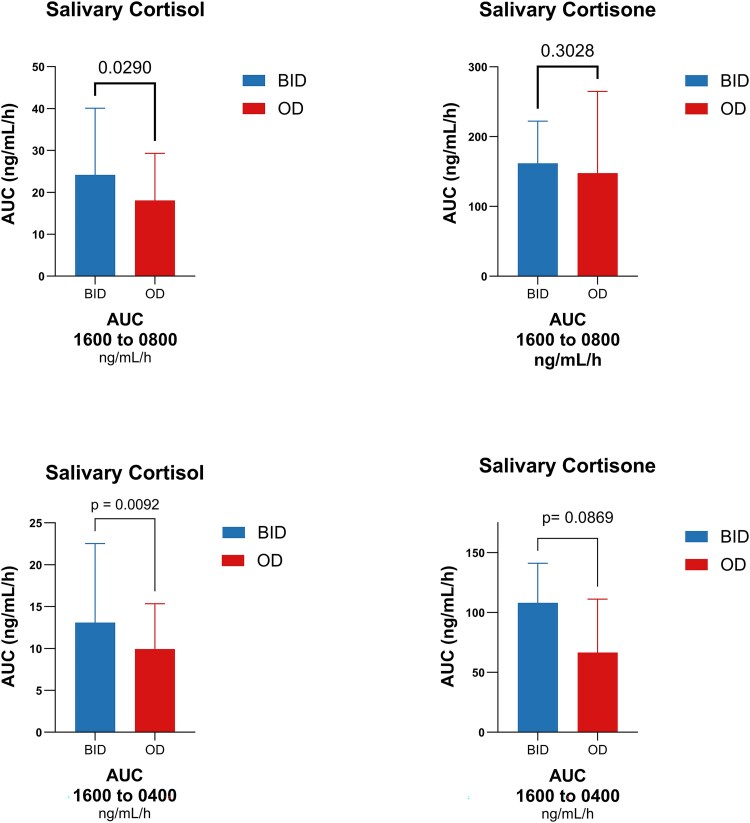
Change in the exposure to cortisone and cortisone in afternoon through early morning (1600-0800 and 1600-0400) measured as estimated AUC of the daily cosinor-based salivary analysis. Data are mean ± SD.

Figure 1 illustrates the salivary cortisol curve estimated by cosinor analysis under T_BID_ (baseline, twice-daily) and T_OD_ (intervention, once-daily). After 2 months of T_OD_, the AUC for cortisol exposure during the 16:00 to 08:00 timeframe significantly decreased (AUC_16:00-08:00_ 18.9 [14.7-32.2] vs 13.6 [11.0-21.6] ng/mL/h, *P* = .029). A similar significant reduction was observed for the 16:00 to 04:00 timeframe (AUC_16:00-04:00_ 13.1 [8.6-22.5] vs 9.9 [6.1-15.3] ng/mL/h, *P* = .009), indicating a drop in cortisol exposure during the late afternoon to early morning (Figs. 1 and 2).

For salivary cortisone, similar but nonsignificant reductions in AUC were observed (Fig. 2). Cortisol and cortisone mesor, acrophase and nadir levels did not change at T_OD_, and acrophase showed a nonsignificant shift to later in the morning. Cortisol and cortisone nadir time remained stable across timepoints (cortisol 21:03 [95% CI: 18:04-00:33] vs 21:03 [95% CI: 16:46-01:19], *P* = .995; cortisone 21:45 [IC95 15:41-02:16] vs 00:00 [IC95 22:32-02:22], *P* = .320). Interestingly, the peak-to-trough ratio of salivary cortisone increased at T_OD_ (3.6 [2.1-6.9] vs 7.8 [3.2-13.3], *P* = .033). (Table 2)

### Changes in Serum and Urinary Steroid Analysis With Once-Daily Osilodrostat Administration

**Figure 3.**
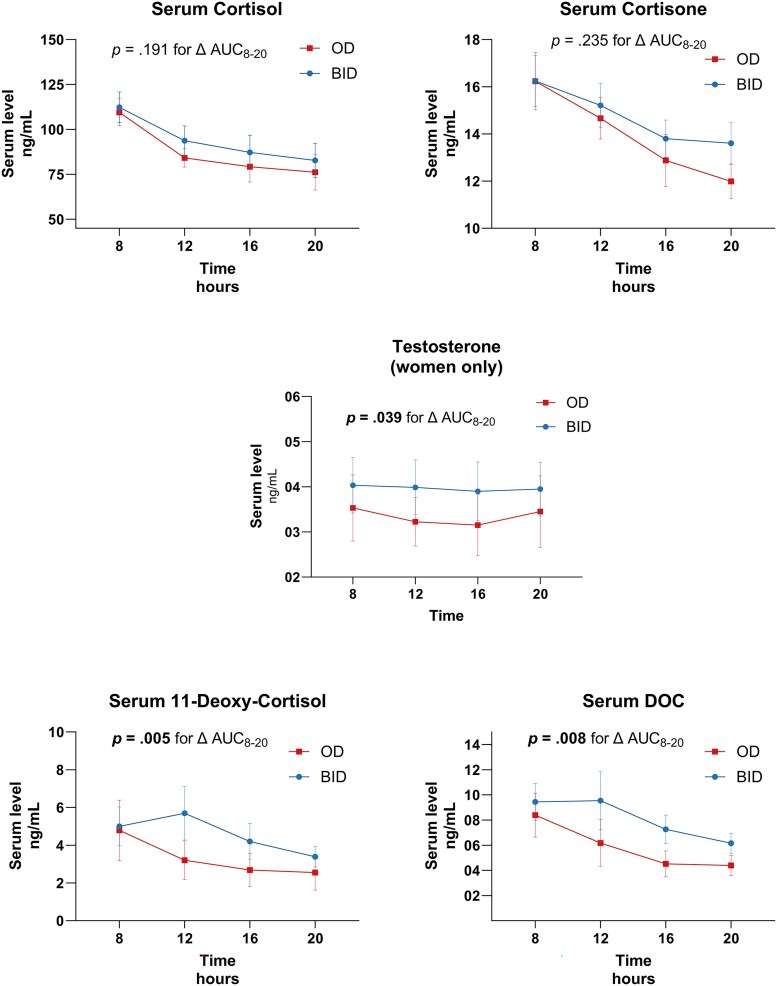
Change in serum steroid day curves and overall exposure (AUC8-20) across T_BID_ and T_OD_. Data are mean ± standard error of the mean.

The changes in serum steroid day curves are illustrated in Fig. 3 and summarized in Table 2. Under T_OD_, serum day curves (08:00-20:00) for cortisol and cortisone showed similar levels at 08:00 but a more pronounced, albeit nonsignificant, decrease throughout the day. No significant changes were observed in overall day exposure for cortisol (cortisol AUC_08:00-20:00_ 1040.4 [795.5-1331.6] vs 937.4 [767.4-1153.0] ng/mL/h, *P* = .191) or cortisone (AUC_08:00-20:00_ 175.7 [IC95 158.5-192.9] vs 166.6 [IC95 146.6-186.6] ng/mL/h, *P* = .235).

Serum steroid precursors demonstrated significant reductions with T_OD_, specifically for 11-deoxycorticosterone (AUC_08:00-20:00_ 8.47 [7.39-12.46] vs 5.25 [2.71-10.41] ng/mL/h, *P* = .008) and 11-deoxycortisol (AUC_08:00-20:00_ 53.8 [21.41-75.51] vs 22.6 [15.78-32.91] ng/mL/h, *P* = .005). Noticeably, testosterone levels in women also showed a significant decrease (AUC_08:00-20:00_ 4.33 [2.35-7.40] vs 3.51 [2.06-4.96] ng/mL/h, *P* = .039).

The shift to once-daily osilodrostat did not impact on the ratio of UFC levels compared to the upper limit of normal (UFC/ULN = 0.59 [IC95 0.36-0.74] vs 0.55 [IC95 0.34-0.73], *P* = .820), confirming equivalence of efficacy. Similarly, no significant changes were observed in urinary cortisone (10.1 [IC95 7.4-12.7] vs 11.4 [7.0-15.9] µg/24 hours, *P* = .359) and, in women, urinary testosterone (0.93 [IC95 0.51-1.36] vs 0.91 [IC95 0.38-1.43] µg/24 hours, *P* = .998).

### Changes in Cardio-Metabolic Parameters and Patient-Reported Outcomes Following Once-Daily Osilodrostat Administration

After shifting to once-daily osilodrostat, blood pressure levels did not change, with respect to systolic (133 [95% CI 122-145] vs 131 [95% CI 120-141] mmHg, *P* = .669), diastolic (88 [95% CI 80-95] vs 83 [95% CI 77-88] mmHg, *P* = .234) and mean blood pressure levels (104 [95% CI 96-111] vs 98 [95% CI 92-104] mmHg, *P* = .164). Similarly, lipid profile, waist circumference, and HOMA index remained unchanged (Table 3), confirming that patients were already well controlled under the twice-daily regimen.

Table 4 summarizes the changes in patient-reported measures. A significant increase in CushingQoL total score was observed with T_OD_ (43.7 ± 15.9 vs 47.9 ± 17.6, *P* = .029), reflecting improved perceived quality of life in disease-specific domains. Sleep quality also improved significantly, as evidenced by a reduction in the PSQI total score (9.9 ± 4.7 vs 8.2 ± 4.8, *P* = .049).

Notably, significant improvements were observed in PSQI component scores, specifically component 2 (sleep latency: 1.4 ± 1.3 vs 0.9 ± 1.1, *P* = .024) and component 3 (sleep duration: 1.8 ± 1.2 vs 1.1 ± 1.1, *P* = .024), indicating shorter sleep latency and longer sleep duration (Table 4). The improvement in sleep quality (PSQI total score) was significantly correlated with the reduction in late afternoon to early morning salivary cortisol exposure (AUC_16:00-04:00_, ρ = .654, *P* = .015). According to the Global Assessment Questionnaire results, 15 patients (93.4%) expressed a clear preference for a once-daily regimen in terms of overall appeal, ease of use, and future treatment choice.

### Exploratory Analysis of Salivary Circadian Rhythm With Once-daily Osilodrostat at 16:00

Following the initial evaluation of T_OD_'s safety and efficacy, an exploratory analysis was conducted to assess the effects of an earlier administration. The once-daily osilodrostat dosing was shifted from 19:00 ± 1 (T_OD_) to 16:00 ± 1 hour (T_OD2_).

Eight patients (mean age 58.8 ± 11.8 years, 2 male patients) completed this additional evaluation and were included in the T_OD2_ analysis. Reasons for non-inclusion included surgery (3 patients), drug withdrawal (2 patients), and refusal to change dosing time for compliance reasons (3 patients). Among the 8 participants, 3 had CD, 5 had adrenal CS, the median disease duration was 4 (2-8.8) years, and the BMI was 30.8 (29.5-33.0). Similar to the main cohort, the majority of patients had mild CS, and achieved disease control under T_BID_ (mean UFC/ULN 0.6 ± 0.4) with a mean dose of 5 ± 2.5 mg. Osilodrostat dose remained unchanged across T_BID_, T_OD_, and T_OD2_, with only the timing of administration being modified.

The results (Supplementary Table S1 and Supplementary Fig. S1) (55) confirmed a reduction in late afternoon to early morning salivary cortisol at both T_OD_ and T_OD2_ compared to T_BID_. Specifically, cortisol AUC_16:00-08:00_ significantly decreased with T_OD2_ compared to T_BID_ (20.2 [15.0-35.5] ng/mL/h at T_BID_ vs 19.3 [11.8-24.0] ng/mL/h at T_OD2_, *P* = .036), while cortisol AUC_16:00-04:00_ showed a borderline significant reduction (13.1 [8.5-25.4] ng/mL/h at T_BID_ vs 12.2 [8.7-14.0] ng/mL/h at T_OD2_, *P* = .050). No significant differences were observed between T_OD_ and T_OD2_ (*P* = .396 for cortisol AUC_16:00-08:00_ and *P* = .293 for cortisol AUC_16:00-04:00_), and similar nonsignificant trends were noted for salivary cortisone AUCs (Supplementary Fig. S1) (55).

Changes in acrophase, mesor, amplitude, and nadir levels of salivary cortisol and cortisone were not significant between T_OD_ and T_OD2_. However, shifting the timing of osilodrostat had a significant phase effect on cosinor cycles. In this set, the 19:00 T_OD_ administration delayed cortisol acrophase (from 05:58 [03:53-08:04] to 08:46 [07:19-10:12], *P* = .025), as was cortisone acrophase (from 05:30 [02:36-08:23] to 08:38 [06:59-10:17], *P* = .025). Shifting the administration to 16:00 slightly anticipated the acrophase but did not fully restore it to T_BID_ levels (*P* = .043 for cortisol). Cortisone acrophase followed a similar pattern (Supplementary Table S1) (55).

The nadir time at T_OD_ showed a trend toward delay, more evident for cortisone (from 20:10 [15:06-01:13] to 00:14 [21:10-03:18], *P* = .069). With T_OD2_, the nadir times returned to levels similar to T_BID_ for both cortisol and cortisone (Supplementary Table S1) (55). The peak-to-trough ratio of salivary cortisone significantly increased at T_OD_ compared to T_BID_ (3.46 [1.80-5.12] vs 11.73 [0.59-24.05], *P* = .028), but this was not observed at T_OD2_ (3.46 [1.80-5.12] vs 4.71 [2.7-6.71], *P* = .225).

Across the 3 timepoints, UFC remained stable and within the normal range, indicating that osilodrostat total daily dose is the primary determinant of disease control (UFC/ULN 0.5 [0.3-0.9] at T_OD_ and 0.2 [0.1-0.8] at T_OD2_).

### Safety Assessments

Once-daily osilodrostat was generally well-tolerated. Liver safety exams remained stable throughout the study (Table 3). No significant ECG alterations were detected, and QTc intervals stayed within the normal range (data not shown). Although 2 patients under T_OD_ had morning cortisol levels below the lower limit of normal, this was not associated with clinical or biochemical signs of adrenal insufficiency (no symptoms, normal electrolytes, and blood pressure). Additionally, no female patients developed clinical signs of hyperandrogenism.

## Discussion

This pilot study demonstrates for the first time that a once-daily administration of osilodrostat, particularly in the evening, is safe, effective, and capable of restoring a more physiological circadian cortisol profile in patients with Cushing syndrome (CS). Compared to the standard twice-daily regimen, once-daily dosing resulted in significantly reduced late afternoon to early morning cortisol exposure (AUC_16:00-08:00_ and AUC_16:00-04:00_) without altering morning peak levels, reflecting an improved alignment with the natural circadian rhythm of glucocorticoids.

These changes were associated with improved patient-reported outcomes, including significant enhancements in quality of life and sleep, 2 critical aspects often impaired in CS (56-59). The correlation between reduced late-night cortisol exposure and improved sleep highlights the potential of this chronotherapy approach to address key clinical pitfalls of hypercortisolism.

Safety assessments confirmed that once-daily osilodrostat was well tolerated, with no cases of adrenal insufficiency, liver toxicity, or QTc prolongation. Blood pressure, lipid profile, and glucose metabolism showed trends toward improvement, reinforcing the potential cardio-metabolic benefits of restoring circadian cortisol rhythms. Importantly, all patients had well-controlled UFC levels at baseline, ensuring that observed benefits were attributable solely to changes in the timing of administration.

Surprisingly, once-daily dosing also led to a significant reduction in steroid precursors (11-deoxycortisol and 11-deoxycorticosterone) and total testosterone levels in women despite stable day cortisol levels. This suggests that osilodrostat, when administered as a single dose, may exert a broader inhibition of the steroidogenic cascade, possibly through higher peak drug concentrations inhibiting enzymes upstream of CYP11B1, such as CYP11A1 (60, 61). These findings align with previous observations that osilodrostat exhibits dose-overproportional kinetics, with higher single doses potentially affecting additional steroidogenic enzymes (62), usually unaffected at lower peak concentrations. Furthermore, recent reports of prolonged adrenocortical blockade after treatment withdrawal suggest that osilodrostat's in vivo actions are more complex than observed in vitro. The LINC-3 trial first documented this phenomenon, showing a delayed UFC rebound after switching from osilodrostat to placebo (63). In 2023, Poirier et al (43) reported sustained adrenal insufficiency lasting 6 weeks to 9 months in 3 patients who discontinued osilodrostat. Similarly, in 2024, Ferriere et al (44) and Tejani et al (45) described 3 additional cases. This “remnant” effect appeared independent of osilodrostat dose, occurring, for an unpredictable duration, in some but not all patients. Notably, affected patients had low or normal 11-deoxycortisol and low DHEA-S despite elevated ACTH. Given these findings, we speculate that osilodrostat may act through 2 distinct mechanisms: a “canonical,” rapid, reversible CYP11B1 inhibition and a long-term effect, potentially involving broader upstream steroidogenesis inhibition or other yet-to-be-explored mechanisms, such as adrenal cytotoxicity. The well-controlled, stable disease on low-dose osilodrostat, along with a long run-in period and extended observation in our cohort of CS, suggests that our circadian findings are primarily attributable to the canonical effect.

Cortisol rhythm restoration has been largely overlooked as an outcome measure in medical treatment for CS, with most clinical trials focusing on overall cortisol production (UFC). However, a few pioneering studies have explored this aspect. Findling et al (23) and Newell-Price et al (24) highlighted late-night salivary cortisol as a marker of circadian rhythm recovery during pasireotide therapy in 2 large phase 3 trials. While these studies advanced alternative monitoring approaches beyond UFC, a single midnight measurement may not fully capture cortisol fluctuations, providing only a partial snapshot rather than a complete profile. In 2013, van der Pas et al (22) evaluated cortisol rhythm in 17 CD patients undergoing stepwise medical therapy (pasireotide, cabergoline, and ketoconazole), observing improvement in 50% of those with an initially disrupted profile. However, this cannot be considered a pure chronotherapy study, as dosing followed routine clinical practice.

The application of chronotherapy in CS is innovative but underexplored. Previous studies in related fields, such as modified-release hydrocortisone for adrenal insufficiency, demonstrated that aligning glucocorticoid therapy with physiological rhythms reduces metabolic complications and restores immune cell circadian profiles (64, 65). Chronotherapy has also been successfully employed in other conditions, including cancer, cardiovascular disease, and neuropsychiatric disorders (66-68).

In hypercortisolism, however, limited evidence of chronotherapy-based approaches is available. Yoshida et al (69) highlighted the role of multiple salivary cortisol measurements as a tool to optimize daily cortisol rhythm in 6 patients with CS. In this study, metyrapone dosing was adjusted based on cortisol profile, resulting in a more physiological rhythm. Debono et al adjusted metyrapone dosing to align with cortisol circadian rhythm, leading to improvement in inflammatory markers (29). In this pioneering phase 1/2a prospective study, 6 patients with adrenal incidentaloma and mild autonomous cortisol secretion (MACS), naïve to medical therapy, were treated with the steroidogenesis inhibitor metyrapone. The timing of dose administration was tailored based on cortisol and cortisone day curves, achieving a reset circadian rhythm associated with a reduction in interleukin-6 levels (29). Our study builds upon Debono's and Yoshida's findings in several ways. First, it includes a larger cohort, nearly 3 times the size, comprising patients with CD and adrenal CS, who were excluded in Debono's study. Second, we evaluated a different CYP11B1 inhibitor (osilodrostat) and tested its effects at different administration times. Finally, we accounted for changes in quality of life and sleep, identifying an overall positive clinical impact of chronotherapy in CS, extending beyond purely biochemical outcomes. All our patients were also well-controlled on standard medical therapy before the study began. Chronopharmacology analyses were performed after 60 to 90 days following the shift in administration timing, ensuring steady-state conditions and enhancing the applicability of our findings to clinical practice. This steady-state design also explains why significant changes in metabolic or biochemical parameters were not observed.

Our results align with clinical experience reported in a recent case series (70), where a higher evening dose was proposed as a strategy for patients with mild CS. Our study lays the groundwork for a dedicated dataset of patients treated with an individualized chronotherapy approach, as recently advocated (71).

Emerging evidence highlights the disruption of circadian transcriptomics and persistent immune alterations in CS even after biochemical remission (17). Rapid restoration of circadian cortisol rhythms should, therefore, be a therapeutic priority, as it may address lingering morbidity and mortality risks associated with CS (17, 18).

This study has limitations. The small sample size, inherent to pilot studies and the rarity of CS, limits the statistical power and generalizability of our findings. Additionally, the lack of a control group of healthy subjects precludes direct comparisons of circadian dynamics. Since all patients were biochemically controlled, and most of them were treated with relatively low doses, this could naturally limit the generalizability of our findings. Indeed, the efficacy of first-line, once-daily osilodrostat treatment in patients with uncontrolled disease and/or naïve to medical treatment was not assessed. For all the above, at this stage, once-daily osilodrostat chronotherapy should be offered to patients with mild CS that is biochemically controlled. Nevertheless, patients under moderate dosages and 1 patient with severe CS on a high dose of 22 mg safely transitioned to the once-daily regimen, achieving improved cortisol rhythm without experiencing any adverse events. Lastly, although no patients in our study developed adrenal insufficiency, we cannot entirely exclude a potential carryover effect. Yet, the observed circadian improvements—particularly the timed reduction in cortisol exposure from afternoon to early morning—are more likely due to the short-term inhibition of CYP11B1 by osilodrostat rather than other mechanisms of action.

The study's strengths include its use of multi-matrix steroid assessment, providing unprecedented insights on steroids fluctuations under medical therapy that could set the basis for novel monitoring strategies in the management of CS. The study is further reinforced by its execution in specialized centers and its pioneering focus on circadian rhythms in CS treated with osilodrostat.

## Conclusions

Our findings demonstrate that once-daily osilodrostat is a feasible, safe, and well-tolerated treatment option for patients with biochemically controlled CS, effectively restoring circadian cortisol rhythms. By achieving lower evening cortisol exposures, this regimen improves sleep quality and overall quality of life. Over the long term, these changes may translate into potential cardiovascular benefits. These results lay the groundwork for future large-scale, long-term studies to fully explore the potential of chronotherapy approach in the management of CS.

## Data Availability

Upon reasonable request, deidentified participant data and code used in the analyses can be shared with other researchers. Data will be made available after the ethics committee approves a study proposal and a data access agreement is signed. For further information, please contact the corresponding author (andrea.isidori@uniroma1.it).

## Clinical Trial Information

Clinical trial registration number: NCT04374721.

## Abbreviations

ACTH: adrenocorticotropic hormone
AUC: area under the curve
BMI: body mass index
CD: Cushing disease
CS: Cushing syndrome
ECG: electrocardiogram
IQR: interquartile range
PSQI: Pittsburgh Sleep Quality Index
T_BID_: stable twice-daily osilodrostat regimen
T_OD_: once-daily osilodrostat regimen
UFC: urinary free cortisol
UHPLC-MS/MS: ultra-high performance liquid chromatography–tandem mass spectrometry
